# Translations

**Published:** 1881-09

**Authors:** William D. Kempton


### TRANSLATIONS.

BY WILLIAM D. KEMPTON, M.D. D.D.S.

### *Perverted Taste due to Salivary Calculus.*

About two years ago, Mr. B., aet. 50, consulted me on account of his very loose lower incisors. He informed me that for several years he had been suffering from an affliction, which, in spite of treatment by several physicians and visits to various bathing places, still persisted. This affliction, which was attributed by the physicians to disordered digestion, consisted in a disgustingly salty taste that he experienced when partaking of food. He was consequently obliged to have his food prepared almost wholly without salt. He could not eat highly seasoned or salted food, such as herrings or cheese, of which he was very fond, on account of their increasing the salty taste to an unbearable extent. On examination I found the looseness of the teeth to be due to the presence of a considerable deposit of salivary calculus. The question at once presented itself to my mind; does not this deposit unite chemically with the saliva and produce this preverted taste? So I carefully removed the calculus and dismissed the patient. In a few days afterwards he returned and informed me that since I had cleaned his teeth the disagreeable taste had entirely disappeared, and that he enjoyed herrings and cheese as much as ever. It might be thought that some other cause was acting in this case, but the best proof to the contrary was the reappearance of the preverted taste as soon as there was a fresh deposit of Salivary Calculus.

(*Hubschmann in Deutsche Vierteljahrschrift fur Zahnheilkunde fur July*, 1881.)

